# S‐nitrosoglutathione reductase maintains mitochondrial homeostasis by promoting clearance of damaged mitochondria in porcine preimplantation embryos

**DOI:** 10.1111/cpr.12990

**Published:** 2021-01-17

**Authors:** Ying‐Jie Niu, Dongjie Zhou, Xiang‐Shun Cui

**Affiliations:** ^1^ Joint International Research Laboratory of Agriculture and Agri‐Product Safety, the Ministry of Education of China Yangzhou University Yangzhou China; ^2^ Department of Animal Science Chungbuk National University Cheongju South Korea

**Keywords:** GSNOR, mitochondria, porcine, preimplantation embryos, S‐nitrosylation

## Abstract

**Objectives:**

S‐nitrosoglutathione reductase (GSNOR), a protein denitrosylase, protects the mitochondria from mitochondrial nitrosative stress. Mammalian preimplantation embryos are mitochondria‐rich, but the effects of GSNOR on mitochondrial function in preimplantation embryos are not well‐studied. In the present study, we investigate whether GSNOR plays a role in mitochondrial regulation during porcine preimplantation embryo development.

**Materials and Methods:**

GSNOR dsRNA was employed to knock down the expression of GSNOR, and Nω‐Nitro‐L‐arginine methyl ester hydrochloride (L‐NAME), a pan‐NOS inhibitor, was used to prevent protein S‐nitrosylation. Mitochondrial amount and function in embryo development were assessed by performing immunofluorescence staining, Western blot, fluorescent probe and real‐time reverse transcription PCR.

**Results:**

GSNOR knock‐down significantly impaired blastocyst formation and quality and markedly induced the increase in protein S‐nitrosylation. Notably, GSNOR knock‐down‐induced overproduction of S‐nitrosylation caused mitochondrial dysfunction, including mitochondrial membrane potential depolarization, mitochondria‐derived reactive oxygen species (ROS) increase and ATP deficiency. Interestingly, GSNOR knock‐down‐induced total mitochondrial amount increase, but the ratio of active mitochondria reduction, suggesting that the damaged mitochondria were accumulated and mitochondrial clearance was inhibited. In addition, damaged mitochondria produced more ROS, and caused DNA damage and apoptosis. Importantly, supplementation with L‐NAME reverses the increase in S‐nitrosylation, accumulation of damaged mitochondria, and oxidative stress‐induced cell death. Interestingly, autophagy was downregulated after GSNOR knock‐down, but reversed by L‐NAME treatment. Thus, GSNOR maintains mitochondrial homeostasis by promoting autophagy and the clearing of damaged mitochondria in porcine preimplantation embryos.

## Graphical abstract

Mitophagy and mitochondrial biogenesis maintain mitochondrial function and contents via promoting damaged mitochondrial clearance, and production of new and healthy mitochondria. Furthermore, autophagy degrades unnecessary proteins and dysfunctional organelles. However, decrease in GSNOR protein levels by knock‐down of *GSNOR* mRNA induces an increase in protein SNOs and prevents mitophagy and autophagy. Thus, GSNOR knock‐down further induces accumulation of damaged mitochondria, oxidative stress and cell death. These harmful effects could be reversed via treatment with L‐NAME.

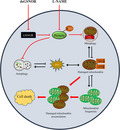

## 1 INTRODUCTION

Mitochondria are key organelles in mammalian preimplantation embryos that supply ATP for most energy‐requiring cellular activities via oxidative phosphorylation. In porcine preimplantation embryos, 91‐97% of the ATP produced is derived from oxidative phosphorylation, with glycolysis making a small contribution (2.6‐8.7% of the total ATP production).^33^ Furthermore, mitochondria also play essential roles in cellular metabolic homeostasis and apoptosis. Numerous studies have indicated that excess production of reactive oxygen species (ROS) in sub‐physical conditions induces mitochondrial dysfunction and compromises preimplantation embryo development.^11^, ^20^ Thus, mitochondrial quality control system is essential for protection from mitochondrial dysfunction and oxidative stress‐induced impairment of embryo development. Damaged mitochondria are cleared via mitophagy, a specific process that degrades membrane potential depolarized mitochondria.^24^

Previous studies have showed that overproduction of reactive nitrogen species, another important redox signal, causes nitrosative stress in mammals.^36^, ^38^ Introducing the nitric oxide group (‐NO) to cysteine thiol in proteins easily forms S‐nitrosothiol (SNO) and has been termed protein S‐nitrosylation.^8^ S‐nitrosylation is catalysed by NO synthases (NOSs), and the enzymes termed S‐nitrosylases.^32^ Physiological S‐nitrosylation modulates the activity of proteins involved in regulating various physiological and biochemical processes in mammalian cells.^9^ Excessive S‐nitrosylation during nitrosative stress has been linked to oocyte ageing, impaired mitochondrial respiratory function and impaired mitophagy.^4^, ^21^ This can trigger protein misfolding, endoplasmic reticulum stress, mitochondrial quality control compromise and mitochondrial dysfunction.^8^ ^,^ ^16^ ^,^ ^27^ Therefore, the NO‐related redox signal may have an important role in the mitochondrial regulation of mammalian oocytes and preimplantation embryos. However, the mitochondrial nitrosative stress and NO‐related redox signal in preimplantation embryos are not well understood.

In mammals, the S‐nitrosoglutathione reductase (*GSNOR*) gene encodes a polypeptide of 385 amino acids with a molecular mass of approximately 40 kDa. Numerous studies have indicated that GSNOR regulates protein S‐nitrosylation by functioning as a protein denitrosylase.^35^ Denitrosylation is considered as an important route for nitrosative stress tolerance. GSNOR expression decreases in primary cells undergoing senescence, as well as in mice and humans during their life span.^28^ GSNOR deficiency causes tumorigenesis^29^ and extensively disrupts cellular homeostasis, including energy metabolism^30^ ^,^ DNA damage repair^39^ and cardiovascular function.^2^ Additionally, GSNOR deficiency causes excessive S‐nitrosylation of dynamin‐related protein 1 (Drp1) and Parkin, impairing mitochondrial dynamics and mitophagy.^28^ Our previous studies have revealed that mitochondrial quality control has an important role in maintaining mitochondrial homeostasis and early embryo development in pigs.^18^ ^,^ ^20^

However, involvement of GSNOR function in NO‐related redox signalling and mitochondrial protection in preimplantation embryos is poorly understood. Therefore, in this study, we employed porcine preimplantation embryos to investigate the effects of GSNOR on mitochondrial amount and function and its underlying mechanisms. First, *GSNOR* mRNA and protein expressions in porcine embryos from the parthenotes to blastocyst stages were assessed. Additionally, GSNOR function in porcine embryonic development was investigated using double‐strand mRNA‐mediated gene knock‐down.

## 2 MATERIALS AND METHODS

Unless otherwise indicated, all chemicals were purchased from Sigma‐Aldrich Co., Inc (St. Louis, MO, USA) and all manipulations were performed on a heated stage adjusted to 38.5°C.

### 2.1 Collection and in vitro maturation of porcine oocytes

All experimental protocols were carried out in accordance with the guidelines of the Institutional Animal Care and Use Committee of the Chungbuk National University Laboratory Animal Center, Cheongju, South Korea.

Ovaries from pre‐pubertal gilts were obtained from a local slaughterhouse (Farm Story Dodarm B&F, Umsung, Chungbuk, South Korea) and transported to the laboratory at 38.5°C in saline supplemented with 75 mg/mL penicillin G and 50 mg/mL streptomycin sulphate. Follicles 3‐6 mm in diameter were aspirated using an 18‐gauge needle connected to a 10 mL disposable syringe. Cumulus‐oocyte complexes were selected based on their morphologic characteristics, that is those showing at least three layers of compact cumulus cells and an evenly granulated ooplasm. After three rinses with in vitro maturation medium TCM‐199 [(11150‐059; Gibco, Grand Island, NY, USA) supplemented with 0.1 g/L sodium pyruvate, 0.6 mmol/L l‐cysteine, 10 ng/mL epidermal growth factor, 10% (v/v) porcine follicular fluid, 10 IU/mL luteinizing hormone and 10 IU/mL follicle‐stimulating hormone], approximately 50 cumulus‐oocyte complexes were transferred to 4‐well dishes (SPL life sciences, Seoul, South Korea) containing 500 μL of the maturation medium. The medium was covered with mineral oil, and the plates were incubated at 38.5°C in a humidified atmosphere containing 5% CO_2_ for 44 hours.

### 2.2 Parthenogenetic activation and in vitro culture

Parthenogenetic activation and in vitro culture were performed as described earlier.^42^ After removing the cumulus cells by repeated pipetting in 1 mg/mL hyaluronidase, denuded oocytes were parthenogenetically activated using 2 direct‐current pulses of 120 V for 60 µs in 297 mmol/L mannitol (pH 7.2) containing 0.1 mmol/L CaCl_2_, 0.05 mmol/L MgSO_4_, 0.01% polyvinyl alcohol (PVA, w/v) and 0.5 mmol/L HEPES. These oocytes were then cultured in bicarbonate‐buffered porcine zygote medium 5 (PZM‐5) containing 4 mg/mL bovine serum albumin (BSA) and 7.5 µg/mL cytochalasin B for 3 hours to suppress extrusion of pseudo‐second polar bodies. Next, the activated oocytes were thoroughly washed and cultured in bicarbonate‐buffered PZM‐5 supplemented with 4 mg/mL BSA in 4‐well plates for 6 days at 38.5°C and 5% CO_2_. Blastocyst formation was examined on day 6 after activation. To determine the total cell number, day 6 blastocysts were randomly collected and stained with 10 mg/mL Hoechst 33342 prepared in PBS for 5 minutes.

### 2.3 GSNOR double‐stranded RNA (dsRNA) preparation

To prepare *GSNOR* dsRNA, *GSNOR* was amplified using a pair of primers Table 1 containing the T7 promoter sequence. The purified PCR products were then used to synthesize dsRNA with a MEGAscript T7 Kit (Ambion, AM1333, Huntingdon, UK) according to the manufacturer's instructions. After in vitro transcription, dsRNA was treated with DNase I and Rnase A to remove the DNA template and any single‐stranded RNA was then purified by phenol‐chloroform extraction and isopropyl alcohol precipitation. The purified dsRNA was dissolved in Rnase‐free water and stored at −80°C until use.

**TABLE 1:** Summary of PCR primers

Gene Name	Accession	Primer Sequence	Product Length
*18S*	NR_046261.1	F: CGCGGTTCTATTTTGTTGGT	219
		R: AGTCGGCATCGTTTATGGTC	
*ND1*	NC_000845.1	F: CCTACTGGCCGTAGCATTCC	162
		R: GAGGATGTGCCTGGTCGTAG	
*GSNOR*	NM_001244833.1	F: GTCATCAAGTGCAAGGCTGC	156
		R: ATCAGCCCCACTCAGGGTAT	
*EGFP*	XM_013480425.1	F: ATGGTGAGCAAGGGCGAGGA	717
		R: CTTGTACAGCTCGTCCATGCCG	
*GSNOR*	NM_001244833.1	F: GATCCTTTGGCCCCTTTGGA	627
		R: TCGGATGCTTTTCCCTGCAT	

### 2.4 Inhibitor preparation and treatment

N_ω_‐Nitro‐L‐arginine methyl ester hydrochloride (L‐NAME, Sigma, N5751) was dissolved in H_2_O to prepare stock solution. To determine whether excess protein S‐nitrosylation was involved in mediating the harmful effects of GSNOR knock‐down in preimplantation embryos, the embryos in PZM‐5 were treated with L‐NAME at 250 µmol/L concentrations. The same amount of H_2_O was added to control oocytes to score the effect of the solvent on the outcome.

### 2.5 Microinjection

For knock‐down experiments, *GSNOR* dsRNA was microinjected into the cytoplasm of a parthenogenetically activated oocyte using an Eppendorf Femto‐Jet (Eppendorf AG, Hamburg, Germany) and Nikon Diaphot ECLIPSE TE300 inverted microscope (Tokyo, Japan) equipped with a Narishige MM0‐202N hydraulic three‐dimensional micromanipulator (Narishige, Inc, Sea Cliff, NY, USA). After injection, oocytes were cultured in PZM‐5. The control group was microinjected with enhanced green fluorescent protein (EGFP) dsRNA.

#### 2.5.1 Biotin‐switched assay for detection of S‐nitrosylated proteins

The biotin‐switch method for detecting S‐nitrosylated proteins was used as described previously.^7^ ^,^ ^13^ In brief, blastocysts from the different experimental groups were fixed in 3.7% paraformaldehyde at room temperature overnight, washed three times with HEN (250 mmol/L HEPES, pH 7.7, 1 mmol/L EDTA, 0.1 mmol/L neocuproine) containing 0.1% Triton X‐100 for 5 minutes. Thiol groups were then blocked with 20 mmol/L methyl methane thiosulphonate (MMTS), a thiol‐reactive agent in the same buffer at 4°C for 30 minutes. The embryos were then washed three times with HEN and incubated with 1 mmol/L ascorbate to reduce the S‐nitrosothiols and with 0.4 mmol/L MTSEA‐Texas Red, a fluorescent derivative of MTSEA in HEN at room temperature for 1 hour. Excess dye was removed by repeated washing of the embryos with HEN containing 0.1% Triton X‐ 100. Stained embryos were then mounted on glass slides in prolonged antifade mounting medium.

### 2.6 ROS measurements

Total ROS levels in blastocysts were determined using 2′,7′‐dichlorodihydrofluorescein diacetate (H_2_DCF‐DA, Cat # D399, Molecular Probes, Eugene, OR, USA) as previously described.^17^ ^,^ ^6^ Briefly, blastocysts were incubated for 15 minutes in phosphate‐buffered saline (PBS)/PVA containing 10 µmol/L H_2_DCF‐DA at 38.5°C. After incubation, blastocysts were washed three times with PBS/PVA. Fluorescence signals were captured as a TIFF file using a digital camera (DP72, Olympus, Tokyo, Japan) connected to a fluorescence microscope (IX70, Olympus). MitoSOX™ mitochondrial superoxide indicator (Thermo Fisher) was then used to evaluate the generation of mitochondrial ROS. Briefly, blastocysts were incubated for 30 minutes in PZM‐5 containing 10 µmol/L MitoSOX™ solution at 38.5°C. After incubation, the blastocysts were washed three times with PBS/PVA and fixed in 3.7% (v/v) paraformaldehyde for 30 minutes at room temperature (20‐25°C). Total and mitochondria‐derived ROS levels were quantified by analysing the fluorescence intensity of the blastocysts using Image J version 1.44g software (National Institutes of Health, Bethesda, MD, USA).

### 2.7 Assay of mitochondrial membrane potential

Live day 6 blastocysts were incubated in PZM‐5 containing 2.5 µmol/L 5,5′,6,6′‐tetrachloro‐1,1′,3,3′‐tetraethyl‐imidacarbocyanine iodide (JC‐1) (Cat # M34152, Invitrogen, Carlsbad, CA, USA) at 38.5°C in 5% CO_2_ for 30 minutes. Fluorescence was visualized using an epifluorescence microscope (Nikon). The membrane potential was calculated as the ratio of red florescence, which corresponded to activated mitochondria (J‐aggregates), to green fluorescence, which corresponded to less‐activated mitochondria (J‐monomers).^34^

### 2.8 Immunofluorescence and confocal microscopy

After washing three times with PBS/PVA, embryos were fixed in 3.7% paraformaldehyde at room temperature for 30 minutes, permeabilized with PBS/PVA containing 0.5% (v/v) Triton X‐100 at room temperature for 30 minutes and incubated in PBS/PVA containing 1.0% (w/v) BSA at room temperature for 1 hour. These embryos were then incubated overnight at 4°C with anti‐GSNOR (1:100; Cat # 11051‐1‐AP, Proteintech), anti‐caspase 3 (1:100; Cat # C8487, Sigma), anti‐γH2A.X (1:100; Cat # 2577, Ser139, Cell Signaling Technology, Danvers, MA, USA), anti‐TOM2O (1:100, F‐10, Cat # SC‐17764, Santa Cruz Biotechnology, Dallas, TX, USA), LC3 (1:100, Cat # 66139‐1‐IG, Proteintech), or BECLIN1 (1:100, Cat # 11306‐1‐AP, Proteintech) diluted in blocking solution. After washing three times with PBS/PVA, the embryos were incubated at room temperature for 1 hour with Alexa Fluor 488^TM^ donkey anti‐mouse IgG (H + L) (1:200; Cat # A21202, Invitrogen), or Alexa Fluor 546^TM^ donkey anti‐rabbit IgG (H + L) (1:200; Cat # A10040, Invitrogen). The embryos were then stained with 10 µg/mL Hoechst 33342 for 10 minutes, washed three times with PBS/PVA, mounted onto slides and examined under a confocal microscope (Zeiss LSM 710 META). Images were processed using Zen software (version 8.0, Zeiss).

To detect total and active mitochondria, blastocysts were firstly incubated with 500 nmol/L MitoTracker Red CMXRos (Cat # M7512, Invitrogen,) at 38.5°C for 30 minutes. After three washes with PZM‐5, immunofluorescence staining of TOM20 was carried out as described above. Total and active mitochondria were quantified by analysing the fluorescence intensity of the blastocysts using Image J version 1.44g software (National Institutes of Health, Bethesda, MD, USA).

### 2.9 Quantitative Reverse Transcription PCR (qRT*‐*PCR)

Blastocysts were collected, and total RNA was extracted from a pool of 30 embryos per group using a TRIzol RNA extraction kit (Invitrogen, USA) according to the manufacturer's instructions. cDNA was obtained by reverse transcription of total RNA using Oligo (dT)_20_ primers and SuperScript III Reverse Transcriptase (Invitrogen). Quantitative PCR was performed by using WizPure™ qPCR Master (Super Green) mix (Cat # W1731‐8, Wizbiosolution, Seongnam, South Korea). Amplification was conducted as follows: 95°C for 3 minutes, followed by 40 cycles of 95°C for 15 seconds, 60°C for 25 seconds, and 72°C for 10 seconds, with a final extension at 72°C for 5 minutes. The target gene was *GSNOR,* with the *18S* gene as the reference. Primers used to amplify each gene are shown in Table 1. mRNA quantities were analysed using the 2^‐ΔΔCT^ method.^14^

### 2.10 MtDNA copy number measurement

Each pool of 10 blastocysts was transferred to a 0.2 mL tube containing 8 μL lysis buffer (20 mmol/L Tris, 0.4 mg/mL proteinase K, 0.9% (v/v) Nonidet‐40, and 0.9% (v/v) Tween 20) at 65°C for 30 minutes, followed by 95°C for 5 minutes. Samples were diluted 1:25 in sterile ddH_2_O before analysis. Subsequently, qPCR was performed as described in the section above.

### 2.11 Western blot analysis

A total of 100 porcine blastocysts per group were lysed with 1 × sodium dodecyl sulphate (SDS) sample buffer by heating at 98°C for 10 minutes. Proteins were separated by SDS‐PAGE and transferred to polyvinylidene fluoride membranes. Next, the membranes were blocked in tris‐buffered saline containing 0.1% (v/v) Tween 20 and 5% (w/v) non‐fat milk for 1 hour and then incubated at 4°C overnight with anti‐GSNOR (1:1000; Cat # 11051‐1‐AP, Proteintech) or β‐tubulin (1:1000; Cat # sc‐5274, Santa Cruz Biotechnology), followed by incubation at room temperature for 1 hour with horseradish peroxidase‐conjugated goat anti‐mouse IgG or goat anti‐rabbit IgG (1:1000; Santa Cruz Biotechnology). Blots were visualized using a CCD camera and UVISoft software (UVITEC Cambridge).

### 2.12 Statistical analysis

Each experiment was repeated at least three times, and representative images are shown in the figures. The GSNOR mRNA and protein expression at different stages were subjected to one‐way analysis of variance. Differences among stages were examined using the Duncan multiple range test. Other data were subjected to the Student's *t* test. Further, all percentage data were subjected to arcsine transformation prior to statistical analysis and then presented as the mean ± standard error (SEM). Significance was set at *P* < .05. All calculations were performed using SPSS software v.19 (SPSS, Inc, Chicago, IL, USA).

## 3 RESULTS

### 3.1 *GSNOR* mRNA and protein expression during porcine preimplantation embryo development

**FIGURE 1:**
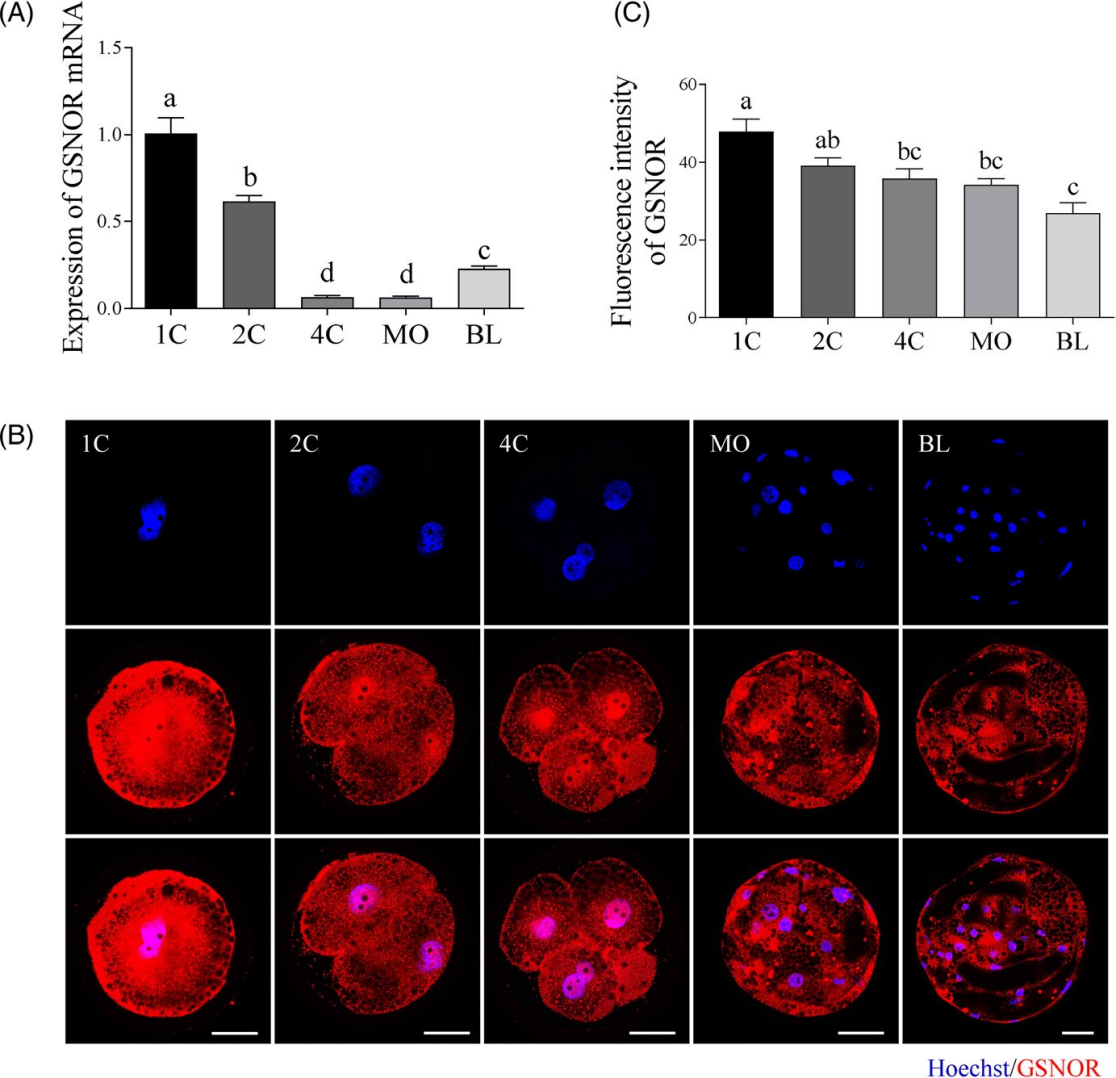
GSNOR mRNA and protein expression during porcine preimplantation embryonic development. A, *GSNOR* mRNA expression levels relative to 1‐cell stage expression level. Immunofluorescence images (B) and relative fluorescence intensity (C) for GSNOR protein expression at 1‐cell (n = 12), 2‐cell (n = 11), 4‐cell (n = 15), morula (n = 16) and blastocyst (n = 14) stages. Scale bars represent 40 μm

First, *GSNOR* mRNA expression was detected using qRT‐PCR. As shown in Figure 1A, the mRNA expression of *GSNOR* was decreased from 1‐cell to 4‐cell embryos and then slightly but significantly increased at the blastocyst stage. We next evaluated the expression and subcellular localization of GSNOR during porcine preimplantation embryo development by immunostaining. GSNOR protein was expressed at all stages in both cytoplasmic and nuclear of preimplantation embryos, but gradually reduced during embryo development Figure 1B,C.

### 3.2 Effects of *GSNOR* knock‐down on porcine preimplantation embryonic development

**FIGURE 2:**
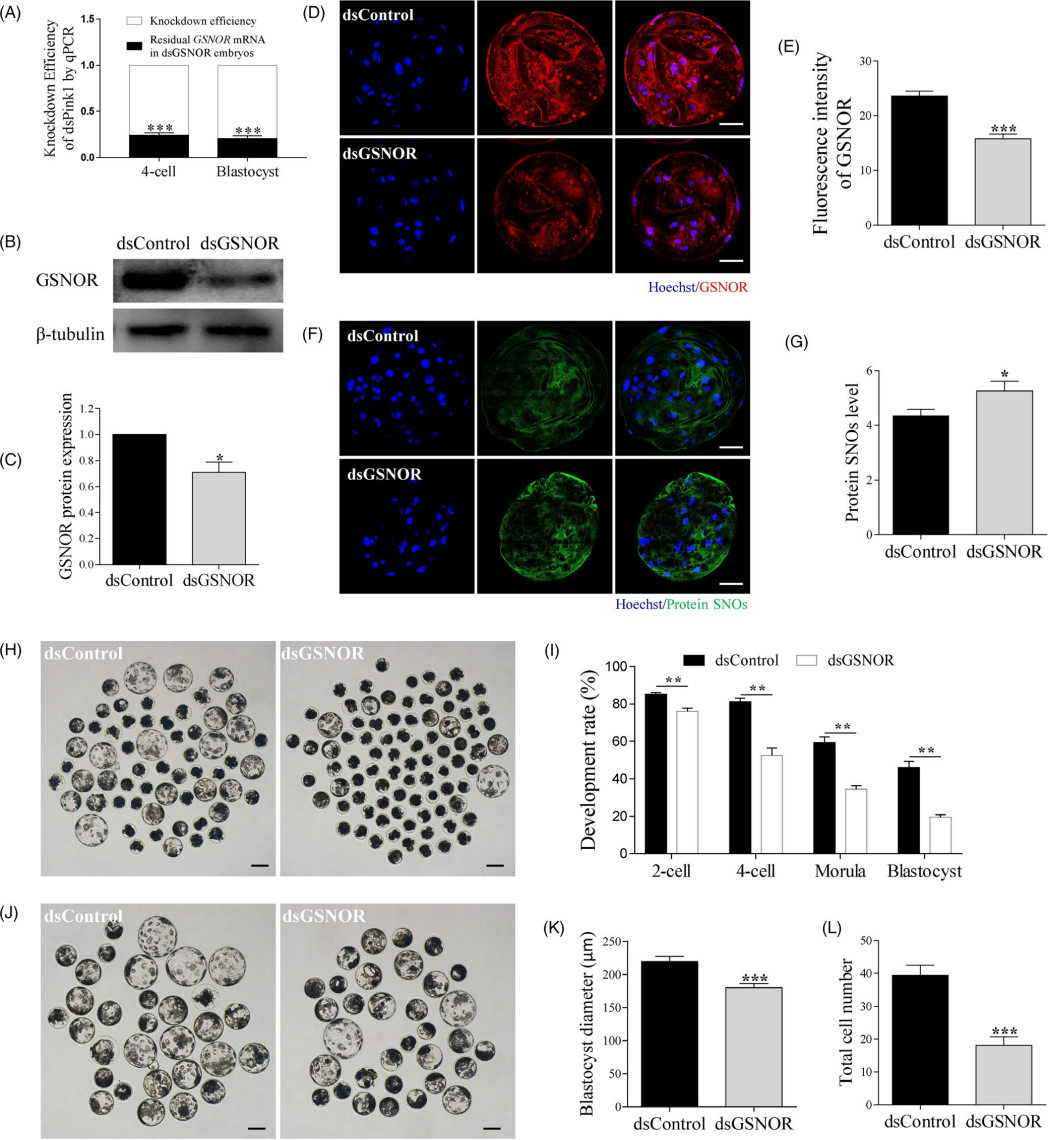
Effects of GSNOR knock‐down on porcine preimplantation embryonic development. A, qRT‐PCR was conducted to confirm *GSNOR* knock‐down at 4‐cell and blastocyst stages following GSNOR double‐stranded RNA (dsGSNOR) injection. *EGFP* dsRNA was injected into porcine parthenotes as the control group (dsControl). B‐E, Western blot and immunofluorescence staining was performed to confirm *GSNOR* knock‐down at blastocyst stage. Scale bars represent 40 μm. Images (F) and related fluorescence intensity (G) comparing protein SNOs in day 6 dsControl (n = 16) and dsGSNOR (n = 18) blastocysts. Scale bars represent 40 μm. The day 6 embryo morphologies (H) and embryo development rate (I) from 2‐cell to blastocyst stages in dsControl and dsGSNOR groups. Scale bars represent 100 μm. Blastocyst images (J) and blastocyst diameter (K) of dsControl (n = 16) and dsGSNOR (n = 17) embryos. Scale bars represent 100 μm. (L) Total cell number of dsControl (n = 32) and dsGSNOR (n = 34) embryos. * (*P* < .05), **(*P* < .01), *** (*P* < .001) vs Control

To examine why GSNOR was expressed at all stages during embryonic development, *GSNOR* double‐stranded RNA (dsGSNOR) was injected into porcine parthenotes, which were then cultured in vitro for 6 days. The knock‐down efficacy on *GSNOR* mRNA was evaluated at the 4‐cell and blastocyst stages. *EGFP* dsRNA was injected into porcine parthenotes as the control group (dsControl). As shown in Figure 2A, compared with the dsControl, *GSNOR* mRNA was effectively downregulated by 76.1% and 79.3% at the 4‐cell and blastocyst stages, respectively (*P* < .001). GSONR protein knock‐down was confirmed by and Western blots Figure 2B,C, *P*< .05, Efficiency: 29.1%) and immunostaining Figure 2D,E, *P* < .001, Efficiency: 33.1%) at blastocyst stages. To detect whether knock‐down of GSNOR caused increased protein S‐nitrosylation, the biotin‐switch assay for detection of S‐nitrosylated proteins was performed.^10^ S‐nitrosylated proteins increased after GSNOR knock‐down compared with control group Figure 2F,G, *P*< .05). Next, the cleavage rate at the 2‐cell and 4‐cell stage, morula and blastocyst formation rate were checked at 24, 48, 96 and 144 hours, respectively. As expected, the 2‐cell cleavage rate (85.1 ± 0.9% vs. 76.1 ± 1.6%, *P* < .01), 4‐cell cleavage rate (81.2 ± 1.9% vs. 52.4 ± 4.0%, *P* < .01), formation of morula (59.3 ± 3.2% vs. 34.5 ± 1.9%, *P* < .01) and formation of blastocysts at day 6 (46.0 ± 3.3% vs. 19.5 ± 1.4%, *P* < .01) were all significantly lower in the dsGSNOR group than those in the dsControl group Figure 2H,I. Additionally, the blastocysts in the dsGSNOR group were smaller than those in the dsControl group Figure 2J. The diameter and total cell number of blastocysts were significantly reduced in dsGSNOR blastocysts compared with those in the dsControl group (219.6 ± 7.8 vs. 180.4 ± 6.0 μm, *P* < .001,39.5 ± 3.0 vs. 18.2 ± 2.5, *P* < .001) Figure 2K,L.

### 3.3 GSNOR knock‐down induces damaged mitochondrial accumulation and mitochondrial ROS production during porcine preimplantation embryonic development

**FIGURE 3:**
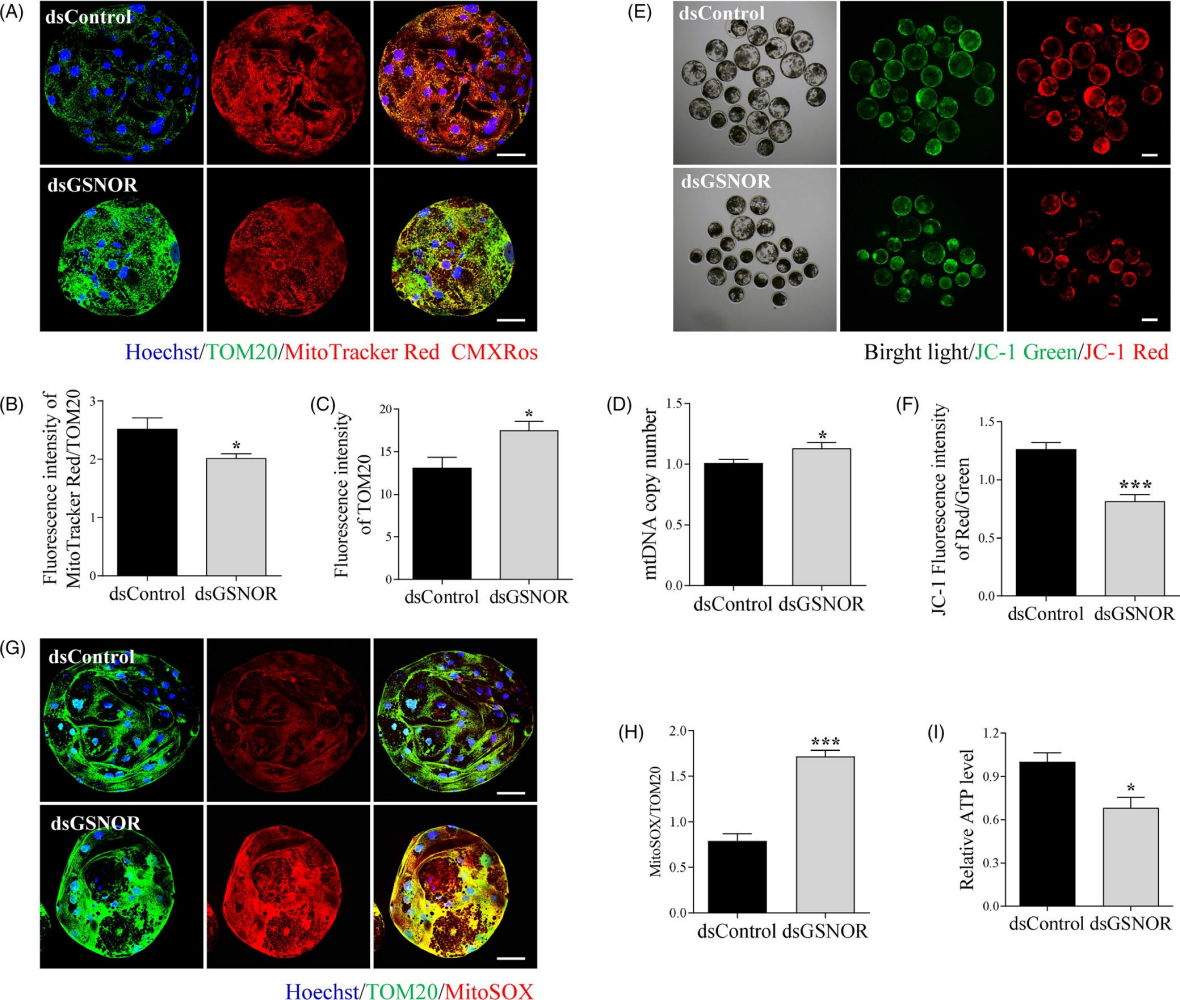
Knock‐down of GSNOR induces accumulation of damaged mitochondria. A, Staining images of TOM20 and MitoTracker Red CMXRos in dsControl and dsGSNOR blastocysts. Scale bars represent 40 μm. Ratio of fluorescence intensity of MitoTracker Red CMXRos to TOM20 (B) and fluorescence intensity of TOM20 (C) in dsControl (n = 21) and dsGSNOR (n = 19) blastocysts. D, Relative mitochondrial DNA copy numbers in dsControl and dsGSNOR blastocysts. JC‐1 staining (E) and mitochondrial membrane potential (F) in dsControl (n = 21) and dsGSNOR (n = 20) blastocysts. Scale bars represent 100 μm. MitoSOX images (G) and mitochondrial ROS levels (H) in dsControl (n = 20) and dsGSNOR (n = 16) blastocysts. Scale bars represent 40 μm. I, ATP levels in dsControl and dsGSNOR blastocysts. * (*P* < .05). *** (*P* < .001) vs Control

Overproduction of S‐nitrosylation causes mitochondrial nitrosative stress and mitochondrial dysfunction. We therefore investigated whether mitochondrial function was compromised in porcine embryos after GSNOR knock‐down. The active and total mitochondria were labelled with MitoTracker Red CMXRos and TOM20, respectively. As shown in Figure 3A‐C, although the fluorescence intensity of TOM20 was significantly increased after GSNOR knock‐down (*P* < .05), the ratio of fluorescence intensity (MitoTracker Red CMXRos/TOM20) was decreased (*P* < .05), indicating total mitochondrial amount increased, but active mitochondria decreased. Moreover, the increase in mitochondrial amount was confirmed by detecting mitochondrial DNA (mtDNA) copy number Figure 3D, *P*< .05). The mitochondrial membrane potential was evaluated by using the JC‐1 fluorescent reaction in porcine embryos. GSNOR knock‐down enhanced green fluorescence and reduced red fluorescence of the JC‐1 dye compared with those in the dsControl Figure 3E. Quantification analysis showed that the ratio of fluorescence intensity (red/green) decreased by nearly 35% in dsGSNOR blastocysts compared with that in the dsControl group, indicating a loss of mitochondrial membrane potential Figure 3F, *P*< .001). Mitochondria‐derived ROS were evaluated in porcine embryos using MitoSOX^TM^ mitochondrial superoxide indicator. As expected, compared with dsControl blastocysts, mitochondria‐derived ROS levels in *GSNOR* knock‐down blastocysts were significantly upregulated by 118% Figure 3G,H, *P* < .001). Finally, the ATP level was significantly reduced in GSNOR knock‐down embryos Figure 3I, *P* < .05). Taken together, in GSNOR deficiency embryos, damaged mitochondria accumulated and caused an increase in ROS, thereby further inducing the mitochondrial membrane potential depolarization and ATP deficiency.

### 3.4 GSNOR knock‐down induces oxidative stress‐derived apoptosis and DNA damage, as well as inhibits autophagic process

**FIGURE 4:**
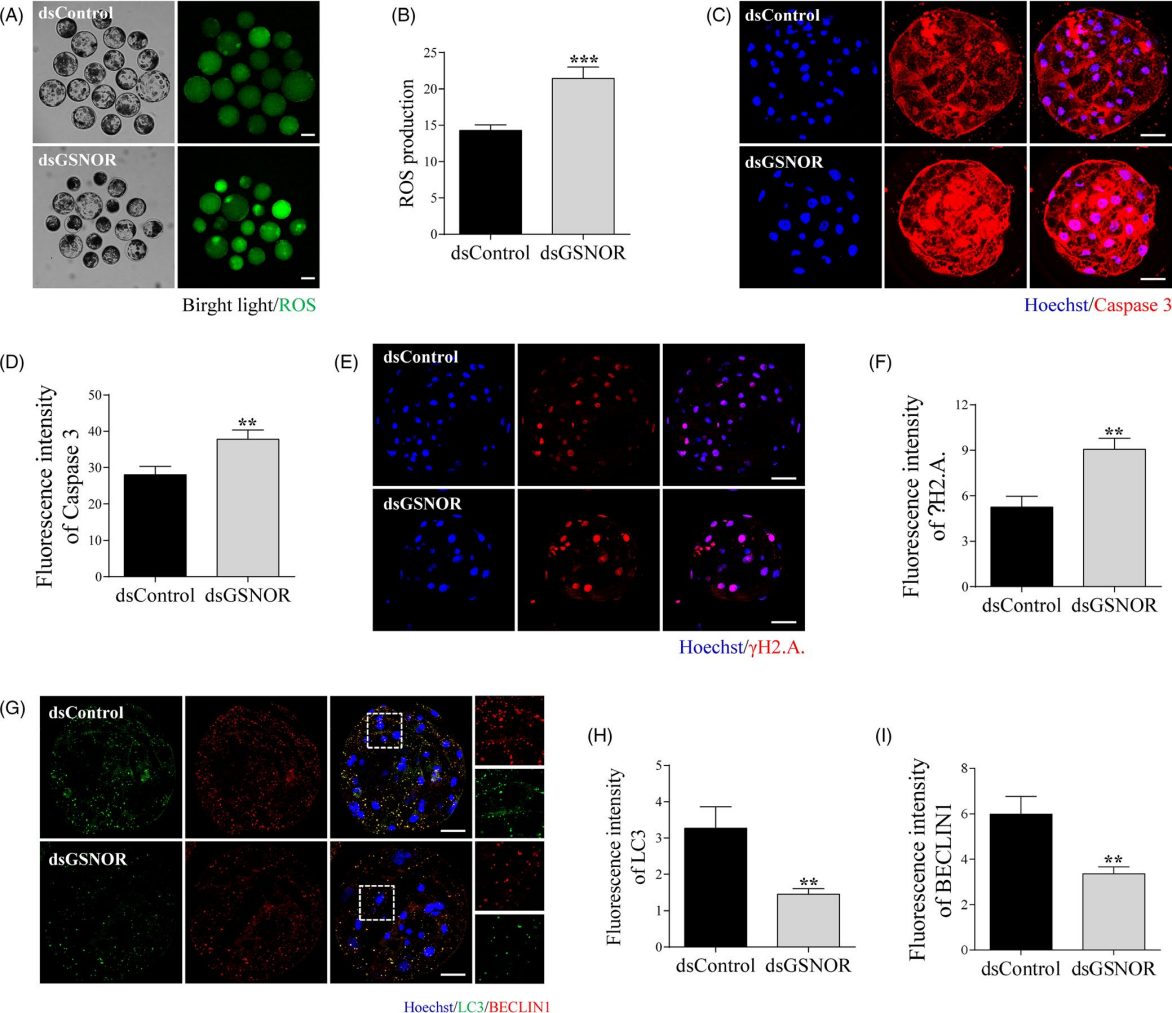
Knock‐down of GSNOR induced oxidative stress‐derived cell death and downregulation of autophagy in porcine embryos. Images (A) and ROS levels (B) in dsControl (n = 18) and dsGSNOR (n = 17) blastocysts. Scale bars represent 100 μm. Images (C) and fluorescence intensity of active caspase 3 (D) in dsControl (n = 17) and dsGSNOR (n = 13) blastocysts. Scale bars represent 40 μm. Images (E) and fluorescence intensity of γH2A.X (F) in dsControl (n = 15) and dsGSNOR (n = 12) blastocysts. Scale bars represent 40 μm. G, Staining images of LC3 and BECLIN1 in dsControl and dsGSNOR blastocysts. Scale bars represent 40 μm. Fluorescence intensity of LC3 (H) and BECLIN1 (I) in dsControl (n = 12) and dsGSNOR (n = 16) blastocysts. **(*P* < .01), *** (*P* < .001) vs Control

Mitochondrial dysfunction was closely linked to excessive intracellular ROS generation, apoptosis, DNA damage and autophagy. Accordingly, total ROS was detected using the H_2_DCF‐DA fluorescent reaction and active caspase 3 was quantified as an apoptosis biomarker. The total ROS and apoptosis were significantly increased by 41% (*P* < .001) and 35% (*P* < .01), respectively, in GSNOR knock‐down blastocysts when compared with control group blastocysts Figure 4A‐D. The effect of GSNOR knock‐down on DNA damage was indicated by levels of γH2A.X in porcine blastocysts Figure 4E,F. Compared with dsControl group, DNA damage in GSNOR knock‐down blastocysts was much higher (*P* < .01). The effect of GSNOR knock‐down on autophagy was detected using BECLIN1 and LC3 staining in porcine blastocysts Figure 4G‐I. As shown in Figure 4G, the number of BECLIN1 and LC3 dots decreased rather than increased in GSNOR knock‐down blastocysts compared with control blastocysts. Quantified data also revealed that the fluorescence intensity of BECLIN1 and LC3 was significantly decreased after GSNOR knock‐down by 44% (*P* < .01) and 55% (*P* < .01), respectively.

### 3.5 L‐NAME prevents GSNOR knock‐down‐induced excessive protein S‐nitrosylation and embryo development impairment

**FIGURE 5:**
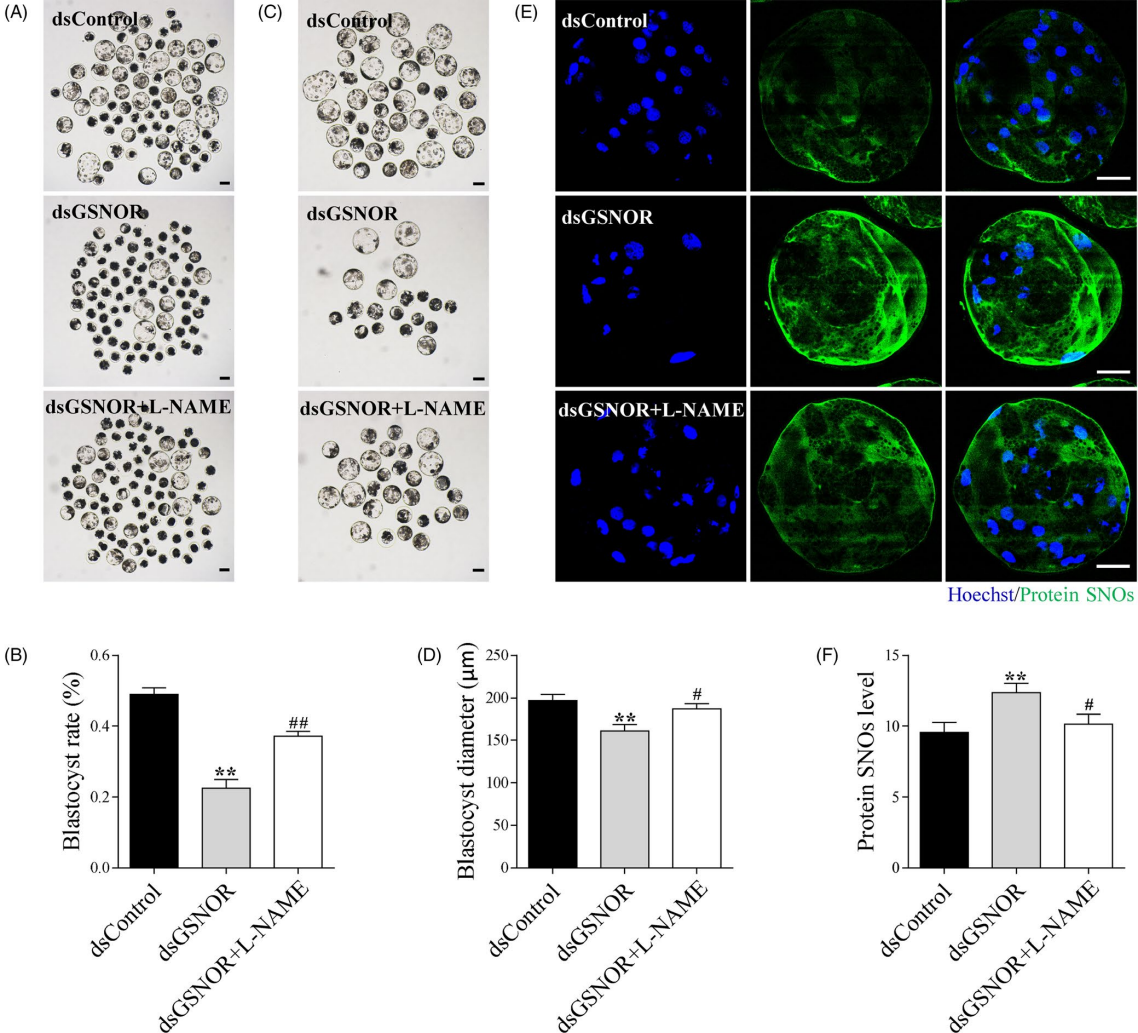
Addition of L‐NAME prevents impairment of embryo development and protein S‐nitrosylation induced by GSNOR knock‐down. The day 6 embryo morphologies (A) and blastocyst rate (B) in dsControl, dsGSNOR and dsGSNOR + L‐NAME groups. Scale bars represent 100 μm. The images of blastocyst (C) and blastocyst diameter (D) in dsControl (n = 41), dsGSNOR (n = 19) and dsGSNOR + L‐NAME (n = 27) groups. Scale bars represent 100 μm. Staining images (E) and related fluorescence intensity (F) comparing protein SNOs expression in dsControl (n = 10), dsGSNOR (n = 13) and dsGSNOR + L‐NAME (n = 11) groups. Scale bars represent 40 μm. **(*P* < .01) vs Control. # (*P* < .05), ## (*P* < .01) vs dsGSNOR

To verify whether the high level of protein S‐nitrosylation induced by GSNOR knock‐down was the main reason for preimplantation embryo development impairments, the pan‐NOS inhibitor, L‐NAME, was added to the medium during in vitro embryo culture. The results indicated that 250 µmol/L L‐NAME could attenuate the SNP‐induced increase in protein S‐nitrosylation level and embryo development impairment and was therefore used for the subsequent study. Compared with the GSNOR knock‐down group, the blastocyst formation rate (49.0 ± 1.5% vs. 22.4 ± 2.1% vs. 37.1 ± 1.2%, *P* < .01) and quality (Blastocyst diameter, 197.2 ± 6.9 vs. 161.0 ± 7.5 vs. 187.0 ± 6.1 μm, *P* < .05) in the dsGSNOR + L‐NAME group was significantly higher, indicating L‐NAME could prevent GSNOR knock‐down‐induced embryo development impairment Figure 5A‐D. In addition, the protein S‐nitrosylation level was evidently decreased after L‐NAME treatment when compared with GSNOR knock‐down group Figure 5E,F, *P* < .05).

### 3.6 L‐NAME prevents GSNOR knock‐down‐induced accumulation of damaged mitochondria, oxidative stress‐derived cell death and downregulation of autophagy in porcine preimplantation embryos

**FIGURE 6:**
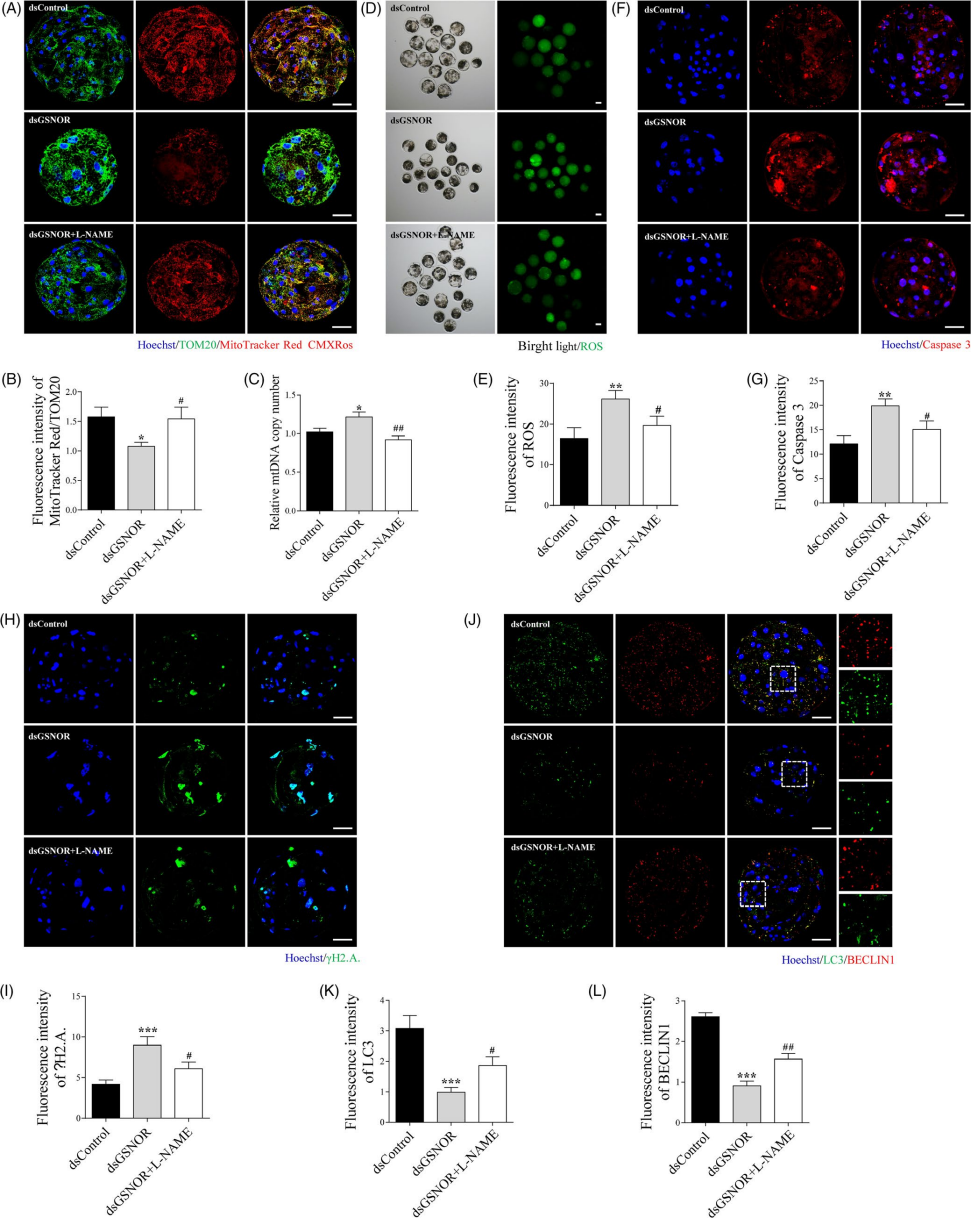
Addition of L‐NAME prevents accumulation of damaged mitochondria, oxidative stress‐derived cell death and downregulation of autophagy induced by GSNOR knock‐down. (A) Staining images of TOM20 and MitoTracker Red CMXRos in dsControl, dsGSNOR and dsGSNOR + L‐NAME blastocysts. Scale bars represent 40 μm. (B) Ratio of fluorescence intensity of MitoTracker Red CMXRos to TOM20 in dsControl (n = 8), dsGSNOR (n = 14) and dsGSNOR + L‐NAME (n = 17) blastocysts. (C) Relative mitochondrial DNA copy number in dsControl, dsGSNOR, and dsGSNOR + L‐NAME blastocysts. Images (D) and ROS level (E) in dsControl (n = 16), dsGSNOR (n = 18) and dsGSNOR + L‐NAME (n = 17) blastocysts. Scale bars represent 100 μm. Images (F) and fluorescence intensity of active caspase 3 (G) in dsControl (n = 12), dsGSNOR (n = 15) and dsGSNOR + L‐NAME (n = 11) blastocysts. Scale bars represent 40 μm. Images (H) and fluorescence intensity of γH2A.X (I) in dsControl (n = 11), dsGSNOR (n = 12), and dsGSNOR + L‐NAME (n = 13) blastocysts. Scale bars represent 40 μm. (J) Staining images of LC3 and BECLIN1 in dsControl, dsGSNOR, and dsGSNOR + L‐NAME blastocysts. Scale bars represent 40 μm. Fluorescence intensity of LC3 (K) and BECLIN1 (L) in dsControl (n = 14), dsGSNOR (n = 15) and dsGSNOR + L‐NAME (n = 17) blastocysts. * (*P* < .05), ** (*P* < .01), *** (*P* < .001) vs Control. # (*P* < .05), ## (*P* < .01) vs dsGSNOR

To further prove whether the high levels of protein S‐nitrosylation induced by GSNOR knock‐down were the main reason for accumulation of damaged mitochondria, oxidative stress‐derived cell death and downregulation of autophagy, L‐NAME was added to the medium during i*n vitro* embryo culture. GSNOR knock‐down‐induced reduction in active mitochondria Figure 6A,B, *P* < .05) and increase in DNA copy number Figure 6C, *P* < .01) were abated after L‐NAME treatment, indicating that the accumulation of damaged mitochondria was rescued. Furthermore, GSNOR knock‐down‐induced ROS production Figure 6D,E, p < .05), active caspase 3 increase Figure 6F,G, p < .05) and γH2A.X expression Figure 6H,I, p < .05) were all rescued by adding L‐NAME, suggesting that oxidative stress‐derived cell death in GSNOR knock‐down group could be rescued by addition of L‐NAME. However, GSNOR knock‐down‐induced autophagy prevention was attenuated after L‐NAME treatment Figure 6J‐L, *P* < .05).

## 4 DISCUSSION

Mammalian oocytes and preimplantation embryos contain a particularly high number of mitochondria. The ROS‐related redox signal has been linked to the mitochondrial dysfunction, and deficiency in ageing oocytes and early embryos exposed to a toxic environment or gene deletion.^3^, ^20^, ^41^ However, little is known about the participation and function of NO‐related redox signals in oocytes and early embryos.^19^, ^22^, ^23^, ^25^ In this study, we first demonstrated that the NO‐related redox signal was involved in preimplantation embryo development. Although GSNOR was highly expressed at 1‐cell stage and then decreased from 4‐cell stage, GSNOR expression was detected at all stage of preimplantation embryos in both cytoplasmic and nuclear, indicating GSNOR and NO‐related redox signals may have important roles during preimplantation embryo development. Moreover, high expression of GSNOR at early cleavage stages many suggested that NO‐related redox signals are also involved to early cleavage or oocyte maturation events. Further studies are needed to detect these detail events and mechanisms. Here, we knocked down expression of *GSNOR* mRNA with *GSNOR* dsRNA and observed that deletion of GSNOR induced protein S‐nitrosylation and impaired embryo cleavage, compaction, blastocyst formation and quality, indicating that GSNOR expression and NO‐related redox signals are essential during porcine preimplantation embryo development. Previous studies have showed that mitochondrial amount and oxidative phosphorylation are increased in blastocyst stage compared with cleavage stages,^37^ ^,^ ^5^ indicating that mitochondrial function is more important for blastocyst formation. Thus, we focused on blastocyst stage to detect the effects and mechanisms of GSNRO knock‐down on mitochondrial regulation and early embryo development.

The equilibrium between S‐nitrosylation and denitrosylation is important for normal cell activity.^2^ Therefore, expression of nitrosylases and denitrosylases requires a dynamic equilibrium to maintain steady‐state concentration of protein SNOs. Here, the expression of GSNOR, one of main denitrosylases, was knocked down, leading to an increase in protein SNOs. The protein S‐nitrosylation level was clearly enhanced after GSNOR knock‐down, suggesting that NO‐related redox signalling was involved to the preimplantation embryo development.

Mitochondrial morphology and activity are dynamically changed during early embryo development with concurrent mitochondrial quality control.^1^ Immunostaining demonstrated that the total mitochondria amount was increased by GSNOR knock‐down, which was confirmed by detection of mtDNA copy number using qPCR. However, the active mitochondria index decrease, mitochondrial membrane potential depolarization, ATP deficiency and mitochondria‐derived ROS increase were showed after GSNOR knock‐down. These results suggested that damaged mitochondria were accumulating after GSNOR knock‐down. Mitochondrial fusion and fission control mitochondrial morphology, while mitophagy clears damaged mitochondria to protect against harmful effects. Mitochondrial biogenesis is involved at different development stages or conditions to promote new and healthy mitochondrial production. The mitochondrial fission component DRP1, mitophagy related proteins PINK1 and PARKIN, and other mitochondrial proteins could be S‐nitrosylated, and inhibiting these protein functions, and thus, preventing the protection effects of mitochondrial quality control.^21^ ^,^ ^28^ In this study, although the S‐nitrosylation status of specific proteins could not be detected due to limited sample availability, the total S‐nitrosylation level was substantially enhanced, suggesting that accumulation of damaged mitochondria at high nitrosative stress due to S‐nitrosylated modification on components of mitochondrial quality control. Depolarized mitochondria produced more ROS, and this higher superoxide level further induced mitochondrial damage, leading to a vicious cycle, with the whole cell ultimately suffering from oxidative stress. Oxidative stress and mitochondrial dysfunction are usually linked to DNA damage and apoptosis. In this study, DNA damage was indicated by γH2A.X, and apoptosis was indicated by active caspase 3. These were both markedly increased after GSNOR knock‐down. Taken together, these results suggest that GSNOR is involved in maintaining normal mitophagy process,GSNOR knock‐down prevented damaged mitochondrial clearance and led to the accumulation of unhealthy mitochondria in preimplantation embryos. Finally, these damaged mitochondria caused oxidative stress‐induced cell death.

Autophagy is a cell adaptive response that degrades unnecessary proteins and dysfunctional organelles and is always upregulated under oxidative stress.^12^, ^31^ Here, although the autophagy level decreased at high oxidative stress after GSNOR knock‐down in porcine preimplantation embryos, this result is consistent with previous reports in cells.^26^ The main reason for this might be that the function of key proteins in the autophagy process is prevented because the protein was S‐nitrosylated in nitrosative stress conditions.^15^ However, the precise molecular pathways that are involved in autophagy inhibition after GSNOR knock‐down require investigation in future studies.

NOSs use L‐arginine as a substrate for the formation of NO. L‐NAME is a highly effective pan‐NOSs inhibitor and as an analogue of L‐arginine can compete with L‐arginine for NOSs.^40^ Therefore, to investigate whether the increase in protein S‐nitrosylation, and its‐induced accumulation of damaged mitochondria, oxidative stress‐derived cell death, and embryo development impairments were due to denitrosylation downregulation in GSNOR knock‐down embryos, L‐NAME was used to inhibit the S‐nitrosylation process. The results indicated that impairment of blastocyst formation and quality in the GSNOR knock‐down group were rescued via L‐NAME treatment. Moreover, the number of active mitochondria increased and total mitochondrial amount was decreased, indicating that accumulation of damaged mitochondria was also abated with L‐NAME compared with GSNOR knock‐down,NOSs inhibition attenuated GSNOR knock‐down‐induced oxidative stress, DNA damage, and apoptosis. GSNOR knock‐down‐induced downregulation of autophagy was also recued with L‐NAME addition, suggesting that GSNOR could denitrosylate the autophagy related proteins to control the autophagy process.

**FIGURE 7:**
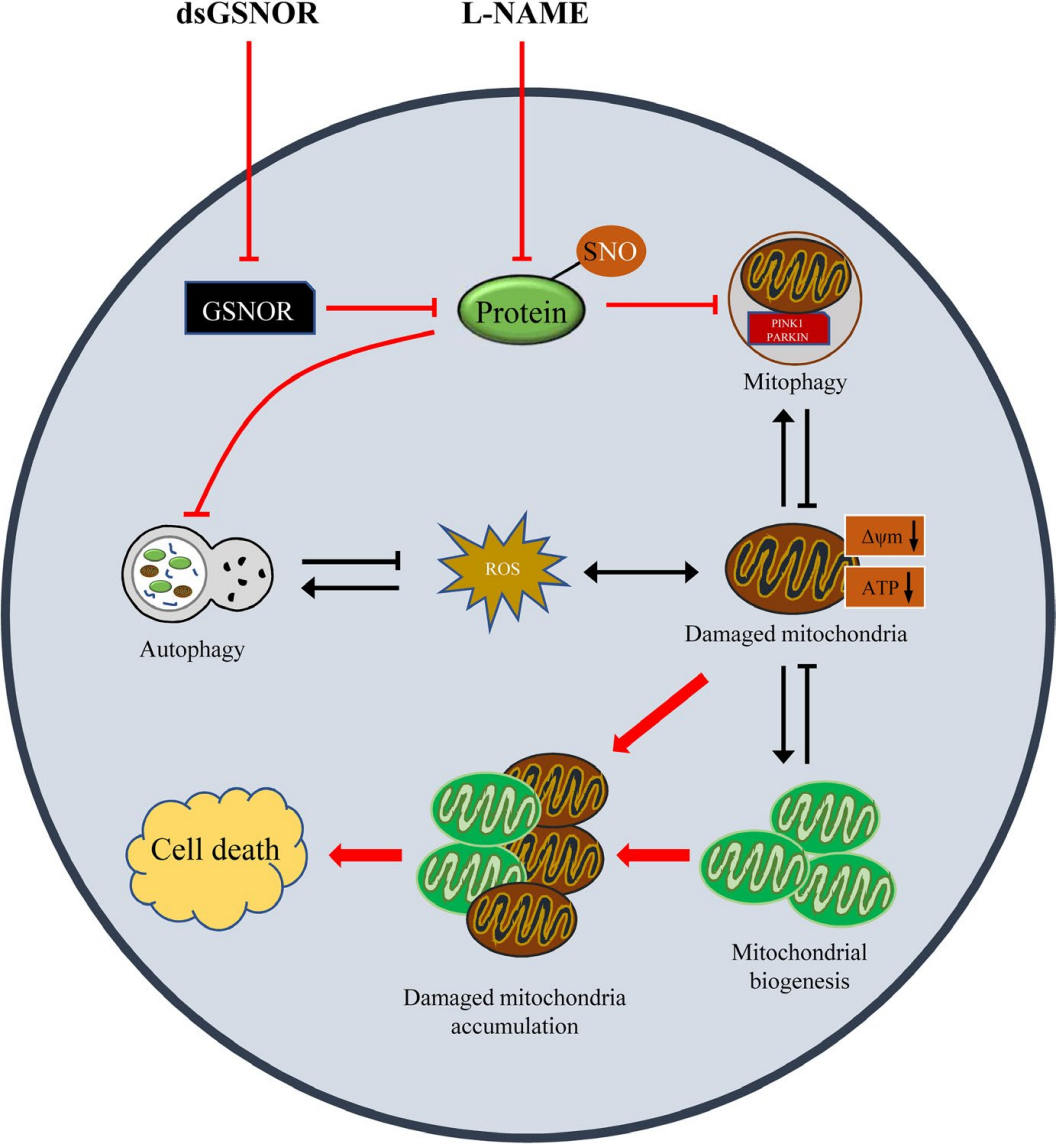
Schematic diagram showing that GSNOR maintains mitochondrial homeostasis by promoting damaged mitochondrial clearance in porcine preimplantation embryos. Normally, mitophagy and mitochondrial biogenesis maintain mitochondrial function and amount via promoting damaged mitochondrial clearance and production of new and healthy mitochondria. Furthermore, autophagy degrades unnecessary proteins and dysfunctional organelles. However, decrease in GSNOR protein levels by knock‐down of *GSNOR* mRNA induces an increase in protein SNOs and prevents mitophagy and autophagy. Thus, GSNOR knock‐down further induces accumulation of damaged mitochondria, oxidative stress and cell death. These harmful effects could be reversed via treatment with L‐NAME. ∆Ψm: mitochondrial membrane potential; ROS: reactive oxygen species. Black arrow indicates the balance between mitophagy and mitochondrial biogenesis and autophagy‐derived protect function. Red arrow indicates the GSNOR knock‐down‐induced increase in protein SNOs, accumulation of damaged mitochondria, downregulation of autophagy and subsequent processes

A schematic diagram Figure 7 demonstrates that when environmental factors or toxins induce mitochondrial dysfunction, depolarized mitochondria can be cleared by mitophagy. Mitochondrial biogenesis can produce new and healthy mitochondria that compensate the deficiency incurred by this. However, GSNOR knock‐down prevents mitophagy and damaged mitochondria keep accumulating in preimplantation embryos. Furthermore, these damaged mitochondria produce more ROS and induce oxidative stress, DNA damage and apoptosis. In addition, GSNOR knock‐down downregulates autophagy and induces a loss of cell adaptive response in the embryos. In conclusion, GSNOR is stably expressed at all stages during porcine embryo development. Protein S‐nitrosylation because of GSNOR knock‐down causes the loss of two important cell protection functions, mitophagy and autophagy, to the embryos.

## CONFLICT OF INTEREST

The authors declare that they have no competing interests.

## AUTHOR CONTRIBUTIONS

Xiang‐Shun Cui and Ying‐Jie Niu designed the experiments. Ying‐Jie Niu and Dongjie Zhou performed the experiments. Ying‐Jie Niu analysed the data and wrote the manuscript. Xiang‐Shun Cui edited the manuscript.

## Data Availability

The data that support the findings of this study are available from the corresponding author upon reasonable request.
